# Migrasomes and tetraspanins in hepatocellular carcinoma: current status and future prospects

**DOI:** 10.2144/fsoa-2023-0086

**Published:** 2023-08-08

**Authors:** Zhongqi Zhang, Tianmiao Zhang, Rongcheng Zhang, Zhengbao Zhang, Shengkui Tan

**Affiliations:** 1 Guangxi Key Laboratory of Environmental Exposomics & Entire Lifecycle Health, Guilin Medical University, Guilin, 541004, Guangxi, China; 2 Department of Epidemiology & Health Statistics, Guilin Medical University, Guilin, 541004, Guangxi, China

**Keywords:** hepatocellular carcinoma, migrasomes, tetraspanins

## Abstract

In recent years, many studies have attempted to clarify the formation, structure and biological function of migrasomes, which are defined as specialized organelles formed by the tips and intersections of Retraction Fibrils during cell migration. It has confirmed that migrasomes were involved in various critical biological processes and diseases, and has became a new research hotspot. In this paper, we reviewed the formation and biological functions of migrasomes, explored the relationship between migrasomes, tetraspanins and hepatocellular carcinoma and discussed the potential applications of migrasomes in hepatocellular carcinoma.

## Plain language summary

Migrasomes are specialized parts of the cell that are responsible for transporting proteins and molecules to their designated cellular locations. In this review, we have attempted to understand their formation, structure and biological function. We have also discussed the potential applications of migrasomes in liver cancer. Our study concluded that more research is required for a better understanding of migrasomes and their contents in liver cancer.

Migrasomes, which were first discovered and reported by Professor Yu and his team at Tsinghua University in 2015, are membranous vesicle-like structures with a diameter of 0.5 to 3 μm [1,2]. During cell migration, long tubular structures called retraction fibrils (RFs) extend from the posterior end [3]. Then, migrasomes form and expand at the tips or intersections of the RFs [1,4–6]. After the RFs break, migrasomes are released in extracellularly and eventually rupture or are taken up by surrounding cells [1]. Up to present, migrasomes are widespread were found in human blood and brain tissue [7–11], a variety of mouse cells and organs [1,11–13], chick cranial neural crest cells [14] and zebrafish embryos [1,5]. Migrasomes were involved in the biological processes of cell communication, embryonic development [5,15], handling of damaged organelles and angiogenesis [12].

Hepatocellular carcinoma is a common and refractory clinical malignancy, the increasing mortality, morbidity and economic burden of which are increased year by year [16]. Therefore, the identification of biomarkers for hepatocellular carcinoma is crucial for the diagnosis and improving the patient's prognosis. As we know, tumor microenvironmental components of hepatocellular carcinoma, such as cytokines, influence tumor immune escape and patient prognosis [17]. Recent studies had also shown that migrasomes and their enriched tetraspanins (TSPANs) were associated with the hepatocellular carcinoma microenvironment and the development of hepatocellular carcinoma [18–22].

In this paper, we have reviewed the formation and biological functions of migrasomes, and the relationship between migrasomes and tetraspanins in hepatocellular carcinoma. Eventually, we believe that migrasomes and their contents have potential applications in hepatocellular carcinoma.

## Formation of migrasomes

### The process of migrasomes formation

Cell migration is the fundamental cellular function that allows multicellular organisms to establish and maintain proper organization. During cell migration, a long tubular structure known as an RF extends along the migration path of the cell [1]. Although RFs were present in various cells, there were limited progression before the discovery of migrasomes [1,3,5,6,23].

The biogenesis of migrasomes can be divided into three stages. First, during the rapid growth phase, migrating cells leave behind a web of RFs as they move forward [1]. Subsequently, migrasomes began to expand at the intersections or tips of the RFs web [1]. As cells continued to move away from the migrasomes, TSPAN4 was transported along the RFs to the interior of the migrasomes, contributing to the spherical shape of the migrasomes [6,24]. After approximately 40 min, the migrasomes reached their maximum size [1]. Second, migrasomes entered the stabilization phase, maintaining their size and structural stability [1]. Finally, as the cells migrated further, the constantly swinging RFs disrupted and caused the migrasomes to be released to the outside of the cell and remain quiescent [1,12]. These migrasomes were either lysed or taken by other cells that migrate to their location [1].

### Elements of migrasomes formation

#### The persistence of cells straight ahead & the speed of cell migration

Cells were moved not exactly in a straight line during migration but changed their direction of migration in varying degrees, with changes in both morphology and polarity [25]. Irregular movement of cells usually occured when the cells were not affected by external factors [26]. When a cell was steered by external factors, the whole body of the cell was elongated and the width of the rear was reduced [27]. While cell changed from a steering process to a straight process the width of cells were rear increasesd, and the number of RFs generation were elevated [27].

According to a study, more RFs were produced when cells changed from the steered state to the straight state, which also means that a greater number of migrasomes could be produced [27]. The study also found that migration rates varied even among the same cells and that cells with faster migration rates could produce longer RFs [27]. Relatively longer RFs have more intersections and tips, providing more growth spots for migrasomes [27]. Hence, RFs not only serve as key channels for transporting cell contents to migrasomes but also provide spots for migrasomes to grow. In more detail, the number and length of RFs are influenced by the persistence and speed of cell migration, which in turn affect the formation of migrasomes.

#### Tetraspanin-enriched macrodomains

TSPANs were consisted of 33 subtypes, a family of proteins containing four transmembrane structures that were widely present in many types of cells [28]. These TSPANs, along with their associated proteins and cholesterol, were distributed laterally on the cell membrane surface, forming tetraspanin-enriched microdomains (TEMs) [29]. TSPANs were also found on the membranes of migrasomes, and the overexpression of 14 of these subtypes enhanced the formation of migrasomes [6]. Among these 14 members, TSPAN9 has the greatest effect on the number of migrasomes formation, and TSPAN4 was the most abundant subtype on the membranes of migrasomes, with a concentration four-times higher than that on RFs [1]. TSPAN4 was aggregatesd into dots on the membranes of RFs, these dots were recruited together with cholesterol on the membranes of migrasomes and aggregate into clusters with other TSPAN proteins to form a domain similar to TEMs [6,24]. Although the composition of this domain was almost the same as that of TEMs, it could reach several microns in the migrasomes membrane, while TEMs were only 100 nm [6]. Therefore, this domain could be said to be composed of several TEMs and was called TEMAs [6].

Huang *et al.* [6] constructed an *in vitro* model of giant unilamellar vesicles (GUV) enriched with TSPAN4, TSPAN7 and cholesterol. The flat GUV film, after applying tension to the it, was stretched into a tubular structure like RFs [6]. At the end of the pulling process, the initially uniformly was distributed TSPAN protein-rich domains spontaneously form TEMAs and undergo an expansion process to become migrasomes-shaped structures [6]. When TSPAN4 was not included in the GUV, no migrasomes-shaped structures were produced, and only cholesterol clusters were distributed along the membrane [6]. This study also measured the relationship between TSPAN4 and cholesterol and GUV bending modulus and found that increasing concentrations of TSPAN4 and cholesterol were positively correlated with the degree of membrane bending [6]. It has also been suggested that the recruitment of TSPAN4 into clusters can reduce the intrinsic curvature of migrasomes, which has a stabilizing effect on the formation of migrasomes and contributes to the stabilization stage of migrasomes [24]. As it stands, the contents of TEMAs are one of the keys to migrasomes formation.

#### Integrins

Integrins, as members of the cell adhesion receptor family, play a role in regulating cell-to-cell and cell-to-extracellular matrix (ECM) junctions. Mammals contain 18 α and 8 β integrins, whereas migrasomes were enriched in α5 and β1 integrins [23,30]. As we all know, these two integrins can both bind specifically to fibronectin.

The migrasomes produced by the cells were not in a highly active state like the contractile filaments [23]. They would adhere at the site of formation and remain almost stationary, which made the cells produce migrasomes that act like anchor points on the constantly moving RFs [23]. Integrins α5 and β1 mentioned above were enriched at the base of globular migrasomes [23]. It was worth noting Wu *et al.* [23] found that the number of migrasomes produced varies in different types of ECM. In fibronectin-rich ECM, integrin β1 and ECM were specifically bound and can produce more migrasomes relative to other types of ECM [23]. Knockout of the integrin α5-encoding gene *ITGA5* in normal rat kidney cells (NRK) also reduced the number of migrasomes generated on fibronectin-rich ECM [23]. Therefore, integrins influence the formation of migrasomes by affecting their adhesion. This phenomenon also raises a question of whether the adhesion of integrins and the mechanical tension generated during cell migration are responsible for the breakage of RFs.

### Composition of the migrasomes

Migrasomes, a type of organelle, have a three-dimensional structure and a diameter ranging from 0.5 to 3 μm, and most migrasomes were ellipsoidal in shape [1]. Similar to other organelles such as lysosomes, endoplasmic reticulum and Golgi apparatus, migrasomes had a single-layer membrane structure and were composed of lipid bilayers [5]. Interestingly, the interior of migrasomes contained monolayer vesicles with diameters ranging from 50 to 100 nm, and the number of vesicles variesd among migrasomes [1]. In addition, migrasomes were enriched in protein composition, with 4737 reproducibly detectable proteins and 577 proteins enriched more than 1.5-fold within the migrasomes compared with the original cells [10]. Migrasomes were enriched in membrane localization proteins, cytoskeletal proteins, cell adhesion proteins, vesicle transport proteins, RNA-binding proteins, chaperone proteins and many other functional proteins [5]. The RNAs were enriched in migrasomes which were predominantly long-stranded (more than 200 nucleotides), and most of its highly enriched RNA were associated with cellular processes such as metabolism, intracellular transport and intercellular signaling [1,4]. It is worth noting that when migrasomes rupture, internal vesicles were released, which contain a large amount of microRNAs that were not present in migrasomes themselves [31]. This also suggests that further research on the composition of migrasomes is still needed.

## Biological function of migrasomes

### Mediating intercellular communication

Currently, migrasomes can only be generated on RFs during cell migration [1] and the cell contents to migrasomes were delivered via RFs [1]. But, when the first cell was left its original position and generated migrasomes, the other cells can move to the migrasomes position and take up the migrasomes [1]. In a study by Zhu *et al.* [4], U87-MG, MDA-MD-468 and PC3 cells caused accumulation of Pten protein (a protein expressed by one of the most abundant full-length mRNAs in the migrasomes) and decreased P-AKT activity in recipient cells after phagocytosis of migrasomes containing full-length Pten mRNA. These suggested that mRNAs and proteins in the migrasomes can be translated by the recipient cells. Thus, the proteins in migrasomes can act directly after uptake by the recipient cells, while the mRNA in migrasomes can play a late regulatory role [4]. This leads us to another type of vesicle that mediates intercellular communication, the exosomes. Exosomes are secreted outward through cell budding or undergo cytokinesis, allowing the release of exosomes outside the cell [32,33]. Subsequently, exosomes interact with target cells and are endocytosed or undergo plasma membrane fusion [32,33]. Therefore, migrasomes, like exosomes, can lead to physiological changes in recipient cells upon reception. However, it remains unknown whether migrasomes, like exosomes, enter recipient cells via endocytosis.

### Embryonic development

During organogenesis in zebrafish embryos, migrasomes were produced in the mesoderm and endoderm of embryonic cells. Migrasomes produced in zebrafish embryos were enriched with chemokines, morphogens, cytokines and growth factors [1,5]. The number of migrasomes produced in TSPAN4a and TSPAN7 deficient mutant zebrafish embryos were significantly reduced, embryonic development was abnormal and the organs of some abnormally developing zebrafish embryos were restored to normal after injection of exogenous migrasomes [5]. It is worth noting that specialized cells called dorsal forerunner cells (DFCs) cluster together during zebrafish embryonic development [5]. Migrasomes accumulated in the cavities formed by DFCs and attracted endodermal cells and DFCs to aggregate [5]. Jiang *et al.* [5] demonstrated that the absence of two chemoattractants, CXCL12A and CXCL12B, in the migrasomes affected DFCs aggregation. Accordingly, it is shown that migrasomes are able to act as specific chemical elicitors, providing specific information to ensure that cells in zebrafish embryos are in the correct spatial position to coordinate the correct occurrence of organs.

Furthermore, in a study of the area of capillary generation in the chick embryo chorioallantoic membrane, monocytes in capillaries produced large numbers of migrasomes, forming migrasome-rich regions [15]. The formation of embryonic capillaries and monocyte aggregation were impaired in the knockdown of TSPAN4 [15]. This also demonstrated the ability of migrasomes to influence embryonic angiogenesis and embryonic development. The current research on the role of migrasomes in embryos of different animal species is not enough, and it suggests that migrasomes may have the potential to maintain normal development of human embryonic organs.

### Removal of damaged organelles

**Figure 1.**
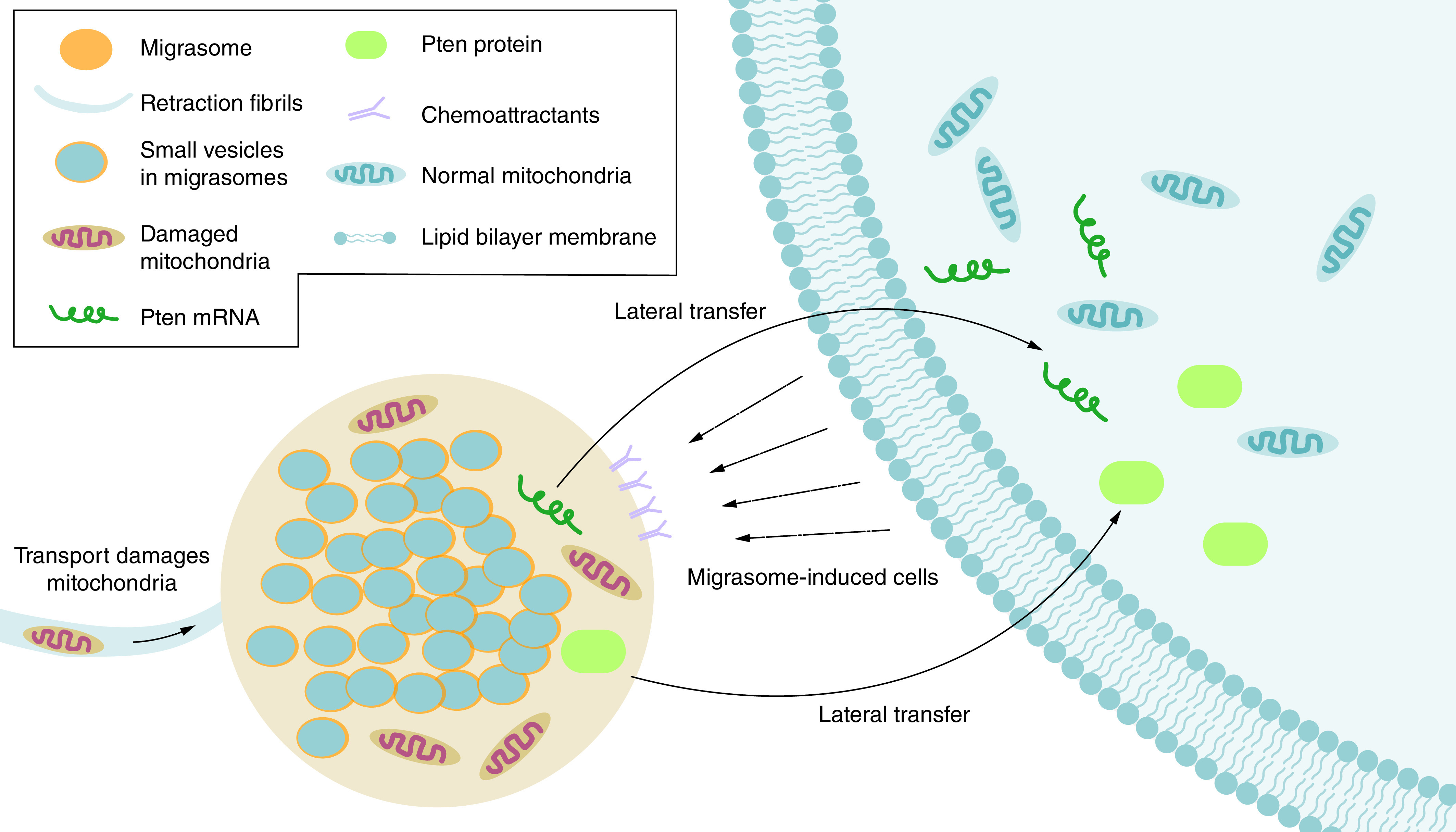
Migrasome and cell during cell migration, migrasomes production at retraction fibrils. Transfer of damaged mitochondria from initial cells to migrasomes. Migrasomes induce other cells to swallow themselves and transfer Pten proteins and Pten mRNA laterally.

Inside the cell, mitochondria are oxidized and cleaved by stress, damage, or dysfunction to remain active and maintain normal cellular respiration. Thus, treatment of mitochondria with stressors induces loss of mitochondrial membrane potential (MMP) and mitochondrial damage. Interestingly, some of the damaged mitochondria accumulated at the base of the cell, entered the RFs, and passed through the RFs to aggregate inside the migrasomes (Figure 1) [12]. As RFs break off, migrasomes were left behind, and damaged mitochondria were removed from the cells [12]. This process was called mitocytosis [12]. The mitocytosis process protected cells from the loss of MMP and mitochondrial respiration induced by mitochondrial stressors and controlled the mass of intracellular mitochondria [12].

It is noteworthy that in the study of Zhu *et al.* [4] proteases could penetrate the membrane of migrasomes and degrade Pten proteins in migrasomes. The membrane of migrasomes was supposed to exist as a protection of the contents from protease-mediated degradation, and we speculated that this function of the membrane of migrasomes might be more convenient for the degradation of their internally damaged organelles.

### Blood vessel formation

We mentioned above that migrasomes were rich in pro-angiogenic factors [15]. In the capillary formation region of the chick embryo chorioallantoic membrane in the study of Zhang *et al.* [15], migrating monocytes were left in areas of migrasomes deposition in the trajectory. These deposited areas of migrasomes were enriched in pro-angiogenic factors, including VEGFA and CXCL12 [15].

As a unique cell population in the body, hematopoietic stem cells have a high capacity for self-renewal and differentiation, and they were also essential for angiogenesis. It had been found that migrasomes produced by multipotent mesenchymal cells (MSCs) could induce KG-1a leukemic cells and CD34 hematopoietic stem cells, and leukemic cells could also take up the migrasomes they produce [34].

Other studies have shown that the pro-inflammatory factor tumor necrosis factor-α (TNF-α) induced cytoskeletal reorganization of endothelial cells in human coronary arteries, enhanced the migratory capacity of endothelial cells in human coronary arteries, and increased the production of migrasomes and angiogenesis [35]. This study suggested that migrasomes act as extracellular signals to promote cell migration.

The possibility that migrasomes were involved in angiogenesis which had been tentatively demonstrated in the above studies, but angiogenesis was not only a complex process in which most cells were in a highly active state but also provided the basis for tumor growth. Accordingly, we believe that the relationship between migrasomes and blood vessel formation should be further explored, especially to establish a link in tumors.

## TSPANs & hepatocellular carcinoma.

Hepatocellular carcinoma is one of the most serious cancers threatening human survival. Among the four major cancers with high mortality rates, hepatocellular carcinoma was the only one whose incidence rate was increasing year by year [36,37]. In addition, it has a high prevalence, especially in developing countries [38]. Therefore, the search for biomarkers of hepatocellular carcinoma is necessary to improve the diagnostic or prognostic situation of hepatocellular carcinoma. Recently, TSPANs have emerged as prominent diagnostic, prognostic markers, and therapeutic targets in neoplasm research. According to a study on the expression of TSPANs in the Oncomine database about hepatocellular carcinoma, the expression of TSPAN7, TSPAN12 and TSPAN28 (CD81) proteins were lower in hepatocellular carcinoma cells than in normal liver tissue [21]. Moreover, overexpression of all three TSPAN proteins enhanced the number of migrasomes produced in NRK cells [6]. Considering the role of migrasomes in cell migration, it is worth examining the effect of TSPANs in migrasomes on hepatocellular carcinoma cells.

### TSPAN1

TSPAN1 was expressed on either the cytoplasm or the cell membrane, and increased the proliferation and invasive ability of many types of cancer cells [22,39,40]. In the study by Chen *et al.* [39], TSPAN1 expression was significantly higher in hepatocellular carcinoma cells compared with paraneoplastic cells and normal human liver cells. In addition,, the expression level of TSPAN1 was positively correlated with the degree of differentiation and clinical stage of hepatocellular carcinoma cells [39]. Patients with high TSPAN1 expression had a lower five-year survival rate, and its overexpression increases the risk of death and affects the prognosis of patients with hepatocellular carcinoma [39].

In the tumor, some of the TSPANs interacted with integrin β1. For example, TSPAN1 and integrin β1 co-localize and interact in intrahepatic cholangiocarcinoma cells [41]. TSPAN24 and integrin β1 amplified PI3K/AKT signaling to induce epithelial–mesenchymal transition (EMT) in hepatocellular carcinoma [42]. Thus TSPAN1 and integrin β1 may play a critical role in hepatocellular carcinoma progression. Surprisingly, Integrin β1 also played an important role in adhesion in migrasomes, and overexpression of both TSPAN1 and integrin β1 enhanced the number of migrasomes produced [31]. We suggest that TSPAN1 may contribute to hepatocellular carcinoma progression by affecting integrin β1.

### TSPAN4

TSPAN4 not only affected the formation of migrasomes but also acted as a marker to effectively differentiate the structure of migrasomes and RFs [1,6,24]. In addition, TSPAN4 was significantly differentially expressed in different cancers [43]. In the current study, TSPAN4 was lowly expressed in lung cancer but highly expressed in hepatocellular carcinoma, gastric cancer and glioblastoma multiforme, and high expression of TSPAN4 was associated with lower survival rates in gastric cancer and glioblastoma multiforme [20,44,45]. These led us to propose the hypothesis that high expression of TSPAN4 may similarly affect survival in hepatocellular carcinoma.

In addition, TSPAN4 expression in hepatocellular carcinoma was highly correlated with tumor-associated macrophages [43]. Implying that TSPAN4 affected the tumor microenvironment of hepatocellular carcinoma. Moreover, there was a correlation between TSPAN4 and cancer treatment-related drugs [19].

Obviously, the expression of TSPAN4 and its impact are not well described in detail in the current studies related to hepatocellular carcinoma. However, TSPAN4 has now demonstrated a potential correlation with hepatocellular carcinoma, and investigating the relationship between TSPAN4 and the prognosis of hepatocellular carcinoma should be a focus of research.

## Migrasomes & hepatocellular carcinoma

Migrasomes are widely present and involved in a variety of diseases. For example, migrasomes served as an early and sensitive indicator of podocyte cell injury [9], ischemia in the brain caused migrasomes production [11], platelets in patients with critical COVID-19 release migrasomes [8] and migrasomes become possible regulators of pancreatic cancer [46]. The association between migrasomes and multiple diseases led us to consider the relationship between migrasomes and the development of hepatocellular carcinoma.

Various cytokines, chemokines and key proteins regulating tumor immunity contained in migrasomes have been demonstrated to have a regulatory role in the tumor immune microenvironment [19,46]. For example, CXCL5 chemokine, which was enriched in migrasomes, promotes proliferation, migration and invasion of hepatocellular carcinoma through activation of the PI3K and ERK1/2 signaling pathways as well as sequential infiltration of neutrophils [47]. Moreover, it was found that CXCL5 was significantly elevated in cirrhotic patients without hepatocellular carcinoma compared with patients with early-stage hepatocellular carcinoma combined with cirrhosis in a clinical study, implying that CXCL5 has the potential to be a test for the development of early hepatocellular carcinoma in cirrhotic patients [48]. Additionally, the migrasomes produced by pancreatic cancer cells are involved in the process of macrophage CD59 expression and immune regulation [46]. This evidence implies that migrasomes and their contents can indeed participate in the tumor microenvironment, with the potential to promote the development of hepatocellular carcinoma.

By analyzing 33 cancer dataset with expression, prognosis, genetic variation and drug sensitivity profiles of genes associated with migrasomes, Qin *et al.* [19] found that the genes ITGB1, ITGA5, EOGT, CPQ, PIGK, NDST1 and TSPAN4 in migrasomes were highly expressed in hepatocellular carcinoma [19]. In the current study, ITGB1 and ITGA5 acted as receptors for the extracellular matrix, and elevated expression of IGTB1 enhances the invasive and proliferative capacity of hepatocellular carcinoma by accelerating the cell cycle process through the PXN/YWHAZ/AKT pathway, whereas ITGA5 was considered to be a prognostic and independent risk factor [49,50]. EOGT is associated with immune infiltration and has the potential to differentiate prognostic markers in patients with hepatocellular carcinoma [51]. SNP 1048575 was associated with low expression of PIGK in patients with hepatocellular carcinoma [52]. These highly expressed genes may serve as genetic markers for hepatocellular carcinoma, suggesting a poor prognosis.

Although the role of TSPANs enriched in migrasomes in hepatocellular carcinoma is not clear at present, the combination of the ability of migrasomes to transport cell contents, mediate intercellular communication, and the survival time of migrasomes in the extracellular matrix up to 200 min, along with the potential prognostic implications of TSPANs in hepatocellular carcinoma, makes it promising as a possible diagnostic marker for hepatocellular carcinoma [1,6]. The ability of migrasomes to participate in angiogenesis may play a key role in the growth of hepatocellular carcinoma [14,15,34].

In conclusion, there were no definitive *in vivo* or *in vitro* studies confirming that migrasomes were found in hepatocellular carcinoma. Current research advances have suggested that migrasomes and its abundant contents should be able to serve as biomarkers for the prognosis and diagnosis of early hepatocellular carcinoma. Furthermore, migrants and their contents are not independent of each other; migrasomes or their contents have been studied independently in past research, but the role of migrasomes contents on the migrasomes themselves should not be overlooked. Therefore, the possible roles of migrasomes in hepatocellular carcinoma and the interactions of various constituent proteins in migrasomes still need to be studied in depth in the future.

## Discussion

Migrasomes have received attention because of their protein enrichment, specific biological functions and disease-related relationships. Migrasomes differ greatly from their morphologically similar extracellular vesicles in terms of their mode of occurrence and protein composition. As a special organelle, the mechanism of migrasomes is more complex, and they have unique functions in embryonic development and clearance of damaged organelles. At this stage, research on the role of migrasomes in hepatocellular carcinoma and other diseases is still in its early stages.

The role played by the TSPANs in hepatocellular carcinoma has received our attention since proteins and mRNAs within migrasomes can be transferred laterally to function in cells and TSPANs are essential components of migrasomes [4,6]. Among them, TSPAN9 was enriched in migrasomes and the overexpression of TSPAN9 had the greatest effect on the number of migrasomes produced [6]. Therefore, we determined the relationship between TSPAN9 expression in immune cell infiltration and immune checkpoints in hepatocellular carcinoma in our previous study [18]. The expression of TSPAN9 in hepatocellular carcinoma was significantly lower than that in adjacent non-hepatocellular carcinoma tissues [18]. Patients with TSPAN9-positive hepatocellular carcinoma had relatively higher overall and disease-free survival and a better prognosis [18]. We found that low expression of TSPAN9 was significantly correlated with the common immune checkpoint CTLA-4 [18]. Implying that high expression of TSPAN9 has a better immunotherapeutic effect [18]. In addition, TSPAN9 was also involved in the formation of TEMs, which were thought to play a key role in the immune microenvironment [18]. The expression of TSPAN9 in hepatocellular carcinoma was negatively correlated with the level of immune cell infiltration, which was also an important factor affecting tumor treatment [18].

Migrasomes, which was originated from cell migration processes, appeared to be a good fit for highly migratory hepatocellular carcinoma. Hepatocellular carcinoma cells achieved cell motility by switching between two migration modes: mesenchymal migration and amoeboid migration, in which cytoskeletal rearrangement-related proteins play a crucial role and migrasomes are rich in cytoskeletal proteins [53], which may provide a clue to the function of migrasomes in the migration process of hepatocellular carcinoma cells. The identification of informative molecules, such as chemokines, that are involved in the correct formation of zebrafish embryos induced by migrasomes remains unknown. In the future, understanding these informative molecules could help us comprehend the specific process of hepatocarcinogenesis.

Integrin αvβ3, an interacting protein with CD147, played a significant role in mediating cell-ECM interactions and transducing of intracellular and extracellular signals during cell adhesion, proliferation, and tumor development [54]. Integrins stabilized pseudopods in hepatocellular carcinoma cells [53], while migrasomes established anchor points through integrin interactions. These similar roles of integrins in both migrasomes and hepatocellular carcinoma suggested a potential link between the two, which merits further investigation.

## Conclusion

In summary, although migrasomes arise only from the migration process of cells, their enriched proteins may not only affect the migration process of hepatocellular carcinoma cells. While there have been no studies linking migrasomes and hepatocellular carcinoma, the available studies suggest that migrasomes and their contents have the potential to influence the progression of hepatocellular carcinoma and serve as a diagnostic and prognostic marker for it.

## Future perspective

The value of migrasomes as a clinical application in hepatocellular carcinoma is starting to emerge, although the pathways through which migrasomes and their enriched proteins contribute to this condition are still not clearly understood. This emerging value is highlighted by the possible role of TSPANs, which are enriched by migrasomes, as a prognostic marker in liver cancer. Additionally, the relationship between TSPANs and the tumor immune microenvironment suggests that migrasomes may also have a role in regulating the tumor microenvironment and the immune system in hepatocellular carcinoma. Thus, migrasomes hold significant potential for future applications in the diagnosis, treatment and prognosis of hepatocellular carcinoma.

**Executive summary**

- The recently discovered organelles called migrasomes are closely linked to cell migration.
- The presence of tetraspanins (TSPANs) in migrasomes have been proved that there were strong association between TSPANs and hepatocellular carcinoma in the current study.

**Formation of migrasomes**

- Migrasomes grow on retraction fibrils (RFs) produced by cell migration.
- The biogenesis of migrasomes can be divided into three stages. First, the rapid growth phase. Second, stabilization phase. Finally, these migrasomes were either lysed or taken by other cells that migrate to their location.
- The persistence of cells straight ahead and the speed of cell migration, tetraspanin-enriched macrodomains and Integrins are elements of the biogenesis of migrasomes.
- Migrasomes, have a three-dimensional structure, and enriched in protein composition.

**Biological function of migrasomes**

- Migrasomes, like exosomes, can lead to physiological changes in the recipient cells upon reception.
- Migrasomes to influence zebrafish embryonic angiogenesis and thus zebrafish embryonic development.
- The Mitocytosis process protected cells from the loss of mitochondrial membrane potential and mitochondrial respiration induced by mitochondrial stressors and controlled the mass of intracellular mitochondria.
- Migrasomes act as extracellular signals to promote cell migration and involve blood vessel formation.

**TSPANs & hepatocellular carcinoma**

- Patients with high TSPAN1 expression have had a lower 5-year survival rate, and its overexpression increases the risk of death and affects the prognosis of patients with hepatocellular carcinoma.
- TSPAN1 may contribute to hepatocellular carcinoma progression by affecting integrin β1.
- TSPAN4 was lowly expressed in lung cancer but highly expressed in hepatocellular carcinoma, high expression of TSPAN4 may associated with lower survival rates in hepatocellular carcinoma.

**Migrasomes & hepatocellular carcinoma**

- Current research advances have suggested that migrasomes and its abundant contents should be able to serve as biomarkers for the prognosis and diagnosis of early hepatocellular carcinoma.
- The possible roles of migrasomes in hepatocellular carcinoma and the interactions of various constituent proteins in migrasomes still need to be studied in depth in the future.

**Discussion**

- While there have been no studies linking migrasomes and hepatocellular carcinoma, the available studies suggest that migrasomes and their contents have the potential to influence the progression of hepatocellular carcinoma and serve as a diagnostic and prognostic marker for it.

**Future perspective**

- We believe migrasomes and their contents can impact hepatocellular carcinoma progression and serve as diagnostic and prognostic markers.
- migrasomes hold significant potential for future applications in the diagnosis, treatment, and prognosis of hepatocellular carcinoma.
